# In Memoriam

**Published:** 1890-08

**Authors:** 


### IN MEMORIAM.

### DR. FREDERICK EHRMANN.

Another pioneer homœopathic physician has passed away. Dr. Frederick Ehrmann died at his residence, No. 56 West Seventh Street, Cincinnati, on June 7th, 1890, in the eighty-third year of his age. He was the son of Dr. Frederick Ehrmann, a physician of some note in Germany. He was born in the village of Jagthausen, in Würtemburg, Germany, on the 18th of February, 1808.

After receiving his education at the University of Tübingen he came to America in the year 1834.

Being convinced of the superiority of the truths of Homœopathy over the prevailing school of medicine, he attended lectures at Allentown, Pa., where instruction was first given by Dr. Hering and others in the new school of medicine. He was the eldest of five brothers, who all became successful homœopathic physicians. He was the last to survive.

After graduating, he settled in Carlisle, Pa., where he practiced his profession for seven years.

It was here that he married Miss Sarah Gibson, niece of Chief Justice Gibson, of Pennsylvania, who, after fifty-three years of married life, lives to mourn his loss.

After a short residence in Baltimore, Md., and Buffalo, N. Y., he removed to Cincinnati, in the year 1850, where he resided and practiced his profession until his decease.

He was among the number who founded the American Institute of Homœopathy, having joined that organization in the year 1846.

In latter years he became an active member of the International Hahnemannian Association, attending most of the meetings until prevented by the infirmities of advancing years.

The Doctor's death came rather unexpectedly, although he had been in feeble health during the past winter. A. H. E.

